# Population Structure of *Phytophthora infestans* from a Single Location in Poland Over a Long Period of Time in Context of Weather Conditions

**DOI:** 10.1007/s00248-020-01630-6

**Published:** 2020-10-29

**Authors:** M. Janiszewska, S. Sobkowiak, E. Stefańczyk, J. Śliwka

**Affiliations:** Plant Breeding and Acclimatization Institute – National Research Institute, Młochów Research Centre, Platanowa 19, 05-831 Młochów, Poland

**Keywords:** Metalaxyl, Microsatellite markers, Late blight, Potato, *Solanum tuberosum*, SSR

## Abstract

*Phytophthora infestans* (Mont.) de Bary is a destructive potato pathogen. Changing weather conditions are among the factors that influence the pathogen population structure. In this study, 237 *P. infestans* isolates were collected from a single unprotected experimental field in an area with high late-blight pressure located in Boguchwała in the southeastern part of Poland during 15 growing seasons (2000–2014). The isolates were assessed for mating type, mitochondrial haplotype, resistance to metalaxyl, virulence, and polymorphism of 14 single-sequence repeat markers (SSRs). The results revealed 89 unique genotypes among the 237 *P. infestans* isolates. Eighty-seven isolates belonged to genotype 34_A1, which was detected in all the years of research except 2012. Isolates of *P. infestans* from individual years were very similar to each other, as shown by Nei’s genetic identity based on 14 SSR markers. The obtained results on isolate characteristics were analyzed in terms of meteorological data (air temperature and precipitation) and indicated that frost, long winters, and hot, dry summers did not directly affect the *P. infestans* population structure. We described the variability in metalaxyl resistance and virulence among isolates of the *P. infestans* genotype 34_A1.

**Supplementary Information:**

The online version contains supplementary material available at 10.1007/s00248-020-01630-6.

## Introduction

Late blight, caused by the oomycete pathogen *Phytophthora infestans* (Mont.) de Bary, is one of the most economically damaging potato (*Solanum tuberosum* L.) and tomato (*Solanum lycopersicum* L.) diseases globally. Under favorable conditions, this pathogen can devastate plants within a week. Most potato cultivars are susceptible to late blight and require intensive chemical protection during cultivation, which increases production cost and has a negative impact on the environment. Yield loss and protection against late blight on average cost approximately six billion USD worldwide [1, 2]. Despite intense efforts to control the disease, *P. infestans* remains a major threat to potato and tomato production [3].

*Phytophthora infestans* is heterothallic, with two mating types named A1 and A2. It can reproduce both asexually and sexually. In asexual reproductive cycles, several hundred thousand sporangia are produced from a single-disease lesion and are dispersed by wind over long distances, causing epidemics. This kind of reproduction supports the development of clonal lineages with minor genetic variation due to mutation and mitotic recombination [4, 5]. Clonal lineages have caused epidemics of late blight, for example, in the USA [6], India [7], and Europe [8]. Sexual reproduction of *P. infestans* is not very common in most regions. Initially, the strains of both mating types occurred only in Mexico, the center of diversity of *P. infestans*. The A2 mating type strains have spread in Europe and other parts of the world since 1980 and were first reported in Switzerland [9, 10]. This migration enabled sexual reproduction of *P. infestans* and, as a consequence, changes in population structure in many regions. Genetically, highly diverse populations have been observed in Denmark, Norway, Sweden, Finland [11, 12], Estonia [13], Russia [14], and Poland [15, 16]. Strains of *P. infestans* resulting from sexual recombination may adapt faster to environmental changes, including the applied fungicides. Another effect of sexual reproduction is the formation of thick-walled oospores that may serve as a source of primary inoculum in the soil, successfully overwintering even in harsh conditions [17].

The constant changes that occur in the structure of *P. infestans* populations around the world include the appearance of new genotypes, differences in the frequencies of genotypes, and shifts between sexual and asexual reproduction. To monitor such changes, *P. infestans* populations are characterized phenotypically and genetically. New molecular techniques that have recently become available, such as next-generation sequencing of whole genomes [18], mitochondrial genome sequencing [19], and genotyping by sequencing (GBS) [20], have enabled insights into the processes forming novel lineages and into pathogen evolution. Nevertheless, 12-plexed single-sequence repeat (SSR) markers have become the most popular tool used for genetic analyses of *P. infestans* population and are currently a global standard for genotyping and classifying lineages. Data on SSR allele frequencies and genotype distributions in populations allow the drawing of conclusions regarding diversity, divergence, gene flow, mutation, and natural selection in the *P. infestans* population structure [20]. Furthermore, SSR markers are a key tool in *P. infestans* monitoring in Europe within the EuroBlight network (www.euroblight.net). Knowledge of the pathogen population structure supports effective late blight management with information, for example, on the pathogen’s ability to overcome host resistance or on resistance to active ingredients of fungicides [21].

One of the most important factors influencing late blight occurrence is weather conditions. Favorable conditions for the development of disease are high air humidity (> 90%) and low temperatures (16 °C) accompanied by rains. Sunlight has a negative impact on the sporulation process; in particular, ultraviolet radiation damages spores [22]. Sporangia are able to survive at temperatures of 3 to 30 °C under high air humidity because the most important factor responsible for reducing their viability is drying [23]. Globally, the average temperatures have risen in recent years, and changes in atmospheric moisture content have been observed, with increasing drought in many regions. All of these climatic changes have an impact on the population structure of *P. infestans*, the survival of genotypes in consecutive years, and the rate of development of late blight disease in the growing season [5, 24].

The aim of this study was to determine the effect of weather conditions on the *P. infestans* population structure that was monitored during 15 growing seasons in one location in Poland. Research included phenotypic and genotypic characterization of Polish *P. infestans* isolates collected in the long term from an unprotected experimental field located on the same farm each year in an area with high disease pressure. Through analyses of data on the diversity of tested isolates in the context of weather conditions during the growing seasons and winters, we aimed to test the hypothesis that cold winters limit the spread of clonal lineages by hampering the survival of mycelia and sporangia while simultaneously promoting diverse, sexually recombined strains of *P. infestans* derived from oospores.

## Materials and Methods

### *Phytophthora infestans* Collection, Isolation of Pure Cultures, DNA Extraction and Storage

Potato leaflets with single late blight lesions were collected during 15 growing seasons (from 2000–2014) from an experimental field in Boguchwała (Podkarpacki Agricultural Advisory Center, Poland). This place is located in the southeastern part of Poland, where climate conditions are favorable for late blight development. Chemical protection against late blight was not applied in the sampled field. The resistance to late blight of potato cultivars and breeding lines grown in this field ranged from susceptibility to high resistance. In total, 237 *P. infestans* isolates were analyzed, of which 116 have already been described by Śliwka et al. [25], Chmielarz et al. [15], and Brylińska et al. [16]. The data were reanalyzed together with new data on 121 *P. infestans* isolates. Due to the small number of samples from 2000 to 2004 (number of isolates: 2000, 4; 2001, 2; 2002, 6; 2003, 5; 2004, 1), the *P. infestans* isolates collected in these years were grouped together and not analyzed in the context of weather data. The number of isolates collected from 2005–2014 is presented in Table 1. The details for each *P. infestans* isolate are listed in Supplementary Table S1. Isolation of pure *P. infestans* cultures was performed using a routine protocol [26]. For long-term storage, the isolates were stored in liquid nitrogen. DNA extraction was carried out as described previously by Brylińska et al. [16]. Total genomic DNA was isolated using a GenElute^TM^ Plant Genomic DNA Miniprep Kit (Sigma-Aldrich, Saint Louis, MO, USA) according to the manufacturer’s instructions. The obtained DNA samples were stored at − 20 °C.

**Table 1:** Number of *P. infestans* isolates collected in 2005–2014 and summer temperature and precipitation in those years (Rzeszów-Jasionka Meteorological Station)

Year	Number of isolates collected	Summer months (June, July, August)			
		Temperature in °C			Amount of precipitation in mm
		Maximal	Average	Minimal	
2005	24	27.7	18.0	11.1	512.1
2006	17	25.2	18.9	10.1	341.5
2007	7	28.0	19.3	13.4	413.6
2008	33	24.2	18.5	13.6	468.1
2009	18	25.8	18.5	10.1	424.3
2010	25	25.8	19.2	11.6	699.4
2011	22	24.8	18.5	12.2	409.4
2012	26	27.5	19.5	12.0	285.5
2013	15	27.6	19.1	12.4	324.0
2014	32	23.8	18.4	11.4	382.8

### Meteorological Data

Air temperature and precipitation data were collected by the Institute of Meteorology and Water Management National Research Institute at the meteorological station in Rzeszów-Jasionka, 16 km from the Podkarpacki Agricultural Advisory Center. The temperature was measured once per hour and precipitation was measured once every 6 h. Weather data from winter 2004/2005 to summer 2014 are presented in Tables 1 and 2.

**Table 2:** Winter temperatures in 2004–2014 preceding *P. infestans* collection (Reszów-Jasionka Meteorological Station)

Season	Winter months (December, January, February)			Number of days with temperature below 0 °C
	Temperature in °C			
	Maximal	Average	Minimal	
2004/2005	11.5	−2.7	−12.5	42
2005/2006	6.5	−1.1	−14.0	45
2006/2007	5.5	−3.7	−24.6	67
2007/2008	10.5	2.1	−8.5	20
2008/2009	9.0	0.6	−10.9	35
2009/2010	7.7	−0.7	−11.2	48
2010/2011	9.8	−3.0	−19.2	63
2011/2012	7.0	−2.6	−12.8	59
2012/2013	6.9	−2.5	−22.3	41
2013/2014	5.3	−2.1	−12.4	59

### Determination of Mating Type and Mitochondrial DNA Haplotype

The mating type was tested for 235 *P. infestans* isolates using PCR markers (W16, S1) and pairing tests. We followed the procedures for mating type identification described previously by Brylińska et al. [27]. The mating type of *P. infestans* isolates collected from 2000–2011 was assessed by a pairing test. From 2009 onward, molecular markers were also used to determine mating types, and after 2011, marker-based identification was the sole method applied.

Mitochondrial DNA haplotypes were identified among 234 *P. infestans* isolates using the PCR primers P2 and P4 as described previously [16, 28]. The products of amplification for the P2 and P4 markers were digested with the restriction enzymes MspI (EC 3.1.21.4) and EcoRI (EC 3.1.21.4), respectively.

### Metalaxyl Resistance and Virulence

The resistance to metalaxyl of 229 *P. infestans* isolates was diagnosed using an in vitro test, according to Bakonyi et al. [29]. Each isolate was plated on rye A agar medium containing metalaxyl (Metalaxyl PESTANAL®; Sigma Aldrich, Saint Louis, MO, USA) at concentrations of 0, 5, and 100 mg L^−1^. An agar fragment containing a mycelium of each tested isolate was placed in the center of two plates for each metalaxyl concentration, and then, the plates were incubated for 13 days in the dark at 16 °C. Radial growth of the mycelium was measured. Isolates were classified as sensitive, intermediately resistant, and resistant as described by Brylińska et al. [16].

The virulence of 226 *P. infestans* isolates was tested using the detached leaflet assay described by Brylińska and Śliwka [30]. This test was performed on 11 Black’s differentials with the genes *R1*–*R11* from *Solanum demissum* (Science and Advice for Scottish Agriculture, Edinburgh, UK) and new resistance sources: the cultivar Bzura with the *R2-like* gene [31]; the cultivar Sárpo Mira with the *R3a*, *R3b*, *R4*, *Rpi-Smira2*, and *Rpi-Smira1* genes [32, 33]; the cultivar Biogold with the *Rpi-abpt* gene [34]; the cultivar Toluca with the *Rpi-blb2* gene [35]; and the diploid breeding lines DG 04-IX-21 with the *Rpi-phu1* gene [36], DG 99-10/36 with the *Rpi-rzc1* gene [37, 38], and DG 99-12/8 with the *Rpi-mch1* gene [39]. A susceptible potato cultivar, Craigs Royal, was used as a control. The inoculum was prepared according to the method described by Sobkowiak and Śliwka [26]. Each differential was tested on two different dates and in two replicates consisting of three lateral leaflets. Not all 229 *P. infestans* isolates have full virulence data sets because some cultivars and breeding lines are new resistance sources that have been included in the virulence assessments later: Sárpo Mira and Biogold since 2006; DG 04-IX-21 and DG 99-10/36 since 2007; DG 99-12/8 since 2009; and Toluca since 2011. For some older isolates, missing virulence data were obtained in 2016 and 2017.

### SSR Marker Analysis

Genotyping of 237 *P. infestans* isolates using 14 SSR loci was performed according to the modified version of the protocol for 12-plex SSR genotyping as described by Li et al. [40]. PCR was conducted using the Qiagen Type-it® Microsatellite PCR Kit (QIAGEN, Hilden, Germany). The volume of the reaction mixture was modified to 12.5 μl and consisted of 6.25 μl of 2× Type-it Multiplex PCR Master Mix, 1.25 μl of a 10× primer mix, 4 μl of PCR-grade water, and 1 μl of template DNA (5–10 ng). The thermal cycling conditions used for the reactions were in accordance with the manufacturer’s protocol for the Qiagen Type-it® Microsatellite PCR Kit. Additionally, two SSR loci were genotyped in a single PCR: Pi33 [41] and G4 [42]. Amplified fragments were analyzed on an automated Applied Biosystems 3500 DNA analyzer (Life Technologies Polska Ltd., Warszawa, Poland). The same 17 *P. infestans* isolates as described by Brylińska et al. [16] were used for calibration of the DNA analyzer. The peak size was determined against a GeneScan^TM^-600 LIZ® Size Standard v2.0 (Life Technologies Polska Ltd., Warszawa, Poland). GeneMapper v.4.0 software (Life Technologies Polska Ltd., Warszawa, Poland) was used to score the fragment lengths of the DNA products of 14 SSR markers.

### Data Analysis

When three alleles at a locus were identified in a *P. infestans* isolate, the middle allele of intermediate size was excluded from analyses based on a diploid model. GenAlex 6.501 software [43] was used to calculate allele frequencies, frequencies of private alleles, Shannon’s Diversity Index, the fixation index, Nei’s [44] gene diversity and genetic differentiation between populations (F_ST_) values. The population structure of the 237 *P. infestans* isolates was investigated by model-based Bayesian clustering carried out in STRUCTURE v.2.3.4 [45]. The data were run using the admixture model, and the cluster numbers (*K*) were evaluated from *K* = 1 to *K* = 20 using 500,000 iterations after a burn-in period of 100,000 iterations. Twenty independent runs at each value of *K* were conducted. The optimal *K* value was estimated using the method described by Evanno et al. [46]. To test evolutionary relationships between genotypes, minimum spanning network (MSN) was constructed and visualized with the *R* package *poppr* [47].

The *P. infestans* genotypes 34_A1 and 13_A2 were identified within the characterized isolates on the basis of their SSR profiles compared with literature data [48] and personal communication with Dr. David Cooke (James Hutton Institute, Dundee, UK).

Differences in frequencies of mating types, mitochondrial haplotypes, sensitivity to metalaxyl, and virulence were tested using Kruskal-Wallis (comparisons between more than two groups) or Mann-Whitney *U* (comparisons between two groups) tests in Statistica 10 software [49].

## Results

### General *Phytophthora infestans* Characteristics

**Fig. 1:**
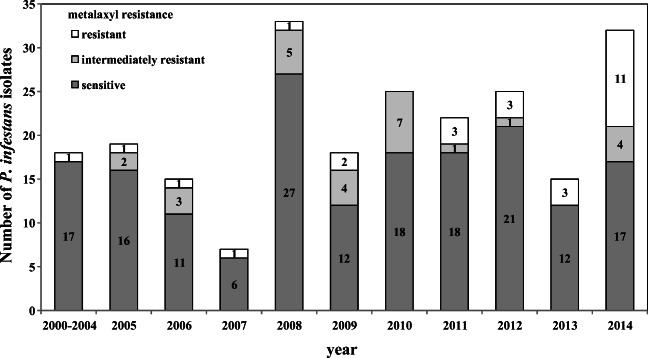
Metalaxyl resistance of 229 *P. infestans* isolates collected in 2000–2014 from the experimental field in Boguchwała

In total, of the 235 *P. infestans* isolates tested, 177 (75%) were of the A1 mating type, and 58 (25%) were of the A2 mating type. Among 234 *P. infestans* isolates, two mitochondrial haplotypes (Ia and IIa) were found, and a majority of the isolates (192 (82%)) were Ia haplotypes, while the other 42 isolates (18%) were IIa haplotypes. In the group of 229 *P. infestans* isolates screened for resistance to metalaxyl, 175 (76%) were sensitive to metalaxyl. The numbers of isolates that were intermediately resistant and resistant to metalaxyl were both equal to 27 (12%). In each year except 2010, from one to three *P. infestans* isolates resistant to metalaxyl were noted (Fig. 1). An increase in the number of resistant isolates to 11 was observed in 2014. The virulence against all 11 Black’s differentials and new resistance sources was tested for 226 isolates. Almost all (99%) of the *P. infestans* isolates were virulent toward plants with the *R1*, *R3*, *R4*, and *R7* genes. Virulence toward plants containing the genes *R10* and *R11* was found in 81% and 92% of the isolates, respectively. Between 51 and 66% of the isolates were able to overcome the resistance conferred by the genes *R2*, *R6*, and *Rpi-mch1* and the resistance of the cultivar Bzura. *Phytophthora infestans* isolates virulent toward plants with the *R8* and *R5* genes and toward the Biogold, Toluca, and Sárpo Mira cultivars were moderately frequent (29–41%). The least frequent isolates were those that were virulent toward plants with the *R9* (12%), *Rpi-phu1* (4%), and *Rpi-rzc1* (0.6%) genes (Supplementary Figure S2). A Kruskal-Wallis test revealed no statistically significant differences in the occurrence of mating type, mitochondrial haplotype, resistance to metalaxyl and virulence factors against 11 Black’s differentials and new resistance sources among the analyzed years (Supplementary Tables S3).

### SSR Genotyping

Fourteen SSR loci were used to genotype the 237 *P. infestans* isolates analyzed in this study. Five isolates were excluded from the analysis because of missing data in more than three loci. In total, 70 alleles were obtained. The number of alleles differed among the loci, ranging from two at loci PinfSSR2, Pi70 and Pi33 to fifteen at locus PiG11 (Table 3). Null alleles were detected at the D13 locus for a large number of isolates (141 isolates). Three alleles per locus were recorded in 108 *P. infestans* isolates at one or more loci. Eleven alleles out of all 70 were detected in only one year of investigation and were called private alleles. Loci D13 and PiG11 had five private alleles each, with frequencies from 0.002 to 0.060. Private allele 246 was noted for PinfSSR6 in 2006, with a frequency of 0.002. Shannon’s Diversity Index of all the isolates treated as one *P. infestans* population was 1.00, ranging per locus from 0.25 (locus Pi70) to 2.10 (locus D13) (Supplementary Tables S4). The fixation index values ranged from − 0.67 (locus Pi04) to 0.56 (locus PinfSSR2) (Supplementary Tables S4).

**Table 3:** Allele frequencies of SSR markers in 237 *P. infestans* isolates collected in Poland from 2000–2014

SSR locus	Allele	Frequency	SSR locus	Allele	Frequency
PinfSSR2	173	0.883	Pi63	270	0.329
	175	0.117		273	0.169
Pi02	258	0.038	G4	161	0.347
	266	0.042		163	0.308
	268	0.871		165	0.345
	270	0.042		276	0.006
	272	0.006		279	0.496
PinfSSR4	285	0.181	PiO4	166	0.463
	287	0.015		168	0.061
	289	0.321		170	0.476
	291	0.034	PiG11	140	0.004
	293	0.100		142	0.050
	295	0.298		148	0.015
	297	0.030		152	0.024
	299	0.021		154	0.026
Pi70	192	0.932		156	0.243
	195	0.068		158	0.054
PinfSSR6	240	0.288		160	0.100
	242	0.193		162	0.317
	244	0.517		164	0.002
	246	0.002		168	0.002
PinfSSR8	260	0.581		198	0.100
	264	0.004		200	0.009
	266	0.415		202	0.041
PinfSSR11	331	0.083		208	0.007
	341	0.755	D13	118	0.179
	356	0.162		122	0.055
Pi4B	205	0.359		132	0.028
	213	0.205		134	0.161
	217	0.436		136	0.193
Pi33	203	0.646		138	0.060
	206	0.354		140	0.005
				148	0.005
				152	0.028
				154	0.211
				156	0.014
				158	0.046
				162	0.018

**Fig. 2:**
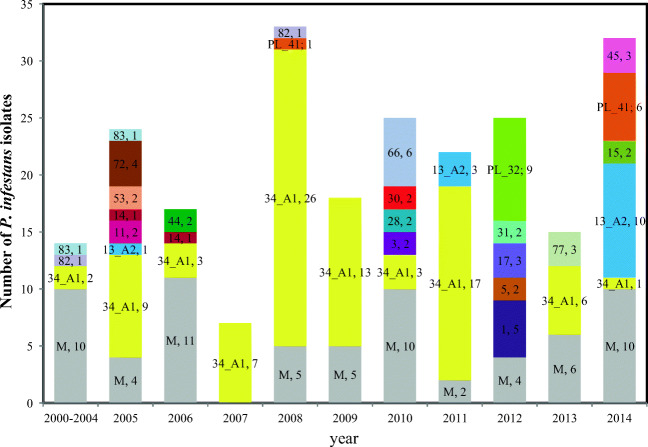
Distribution of *P. infestans* genotypes of isolates collected in 2000–2014 from Boguchwała. Gray indicates miscellaneous genotypes detected in single isolates. Different colors indicate genotypes detected more than once in the population sample. Number tags on the bar show the genotype name and number of isolates of the given genotype

Within the 232 *P. infestans* isolates, 89 unique genotypes were identified, allowing differences of up to two alleles within a genotype. A total of 67 genotypes were represented by only one isolate (Fig. 2). The most frequent genotype 34_A1 was represented by 87 *P. infestans* isolates, was detected for the first time in 2002 (2 isolates), and was present in all years within the 2005–2014 period except 2012. The second most common genotype was 13_A2 (14 isolates in three years of research). Nine isolates of the *P. infestans* genotype PL_32 were detected in 2012. Six isolates of the genotype PL_41 were detected in 2014, and one was found in 2008. Three of the genotypes were found in 2 years and were represented by a single isolate each year. Eleven genotypes were represented by two or three isolates of *P. infestans* in only 1 year of the study (Fig. 2).

A comparison carried out using Nei’s genetic identity based on 14 SSR markers showed that the samples from individual years were very similar to each other, with high Nei’s coefficients (Supplementary Tables S4). The highest Nei’s coefficient (0.97) was between 2008 and 2011, and the lowest (0.72) was between 2007 (all isolates of the genotype 34_A1) and 2012 (no isolates of the genotype 34_A1 found). For these samples, pairwise *F*_*ST*_ values were also calculated, and the highest value of 0.17 was obtained between 2007 and 2012, while the lowest value of 0.02 was obtained between 2008 and 2011 (Supplementary Tables S4). The greatest sample diversity was observed in 2014, with a Shannon’s Diversity Index value of 0.93. In 2007 and 2008, the lowest values of Shannon’s Diversity Index (0.53 and 0.68, respectively) were noted (Supplementary Tables S4).

**Fig. 3:**
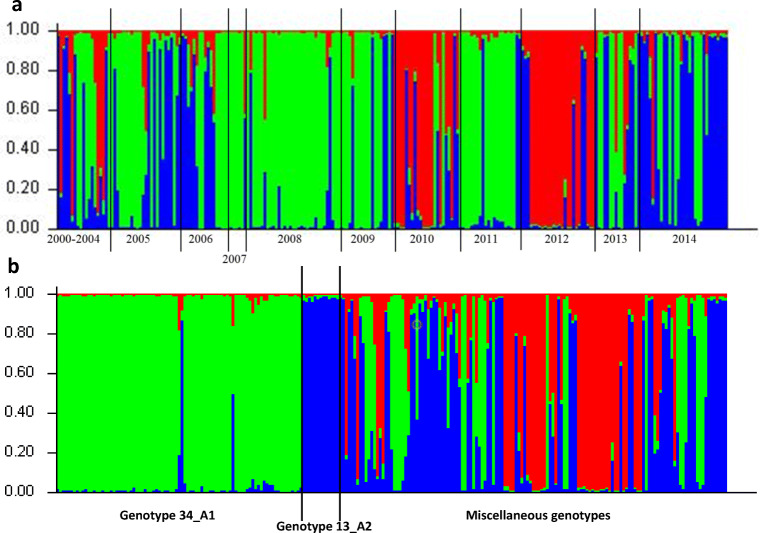
Structure analysis of *P. infestans* isolates collected in 2000–2014 from the Boguchwała location based on 14 simple sequence repeat loci performed with the program STRUCTURE v.2.3.4. Clusters were sorted by year of collection (**a**) and genotypes (**b**)

The genetic population structure of *P. infestans* samples from one experimental field in Boguchwała from 2000–2014 was analyzed using the program STRUCTURE for three clusters (*K* = 3), which were calculated based on the method proposed by Evanno et al. [46]. Isolates were divided into “green,” “blue,” and “red” clusters (Fig. 3a, b). The “red” cluster contained most of the *P. infestans* samples from 2010 and 2012 (isolates of miscellaneous genotypes and genotypes detected more than once in these 2 years) and was rarely present in other years. The “green” cluster contained isolates of 34_A1 genotype (Fig. 3b). Isolates of the 13_A2 genotype and others were in the “blue” cluster represented in all years. We also identified 43 isolates that could not be ascribed to only one cluster. The results of STRUCTURE analysis were also consistent with the other data obtained in our research, such as the distribution of *P. infestans* genotypes (Fig. 2).

### Genotype Descriptions

**Fig. 4:**
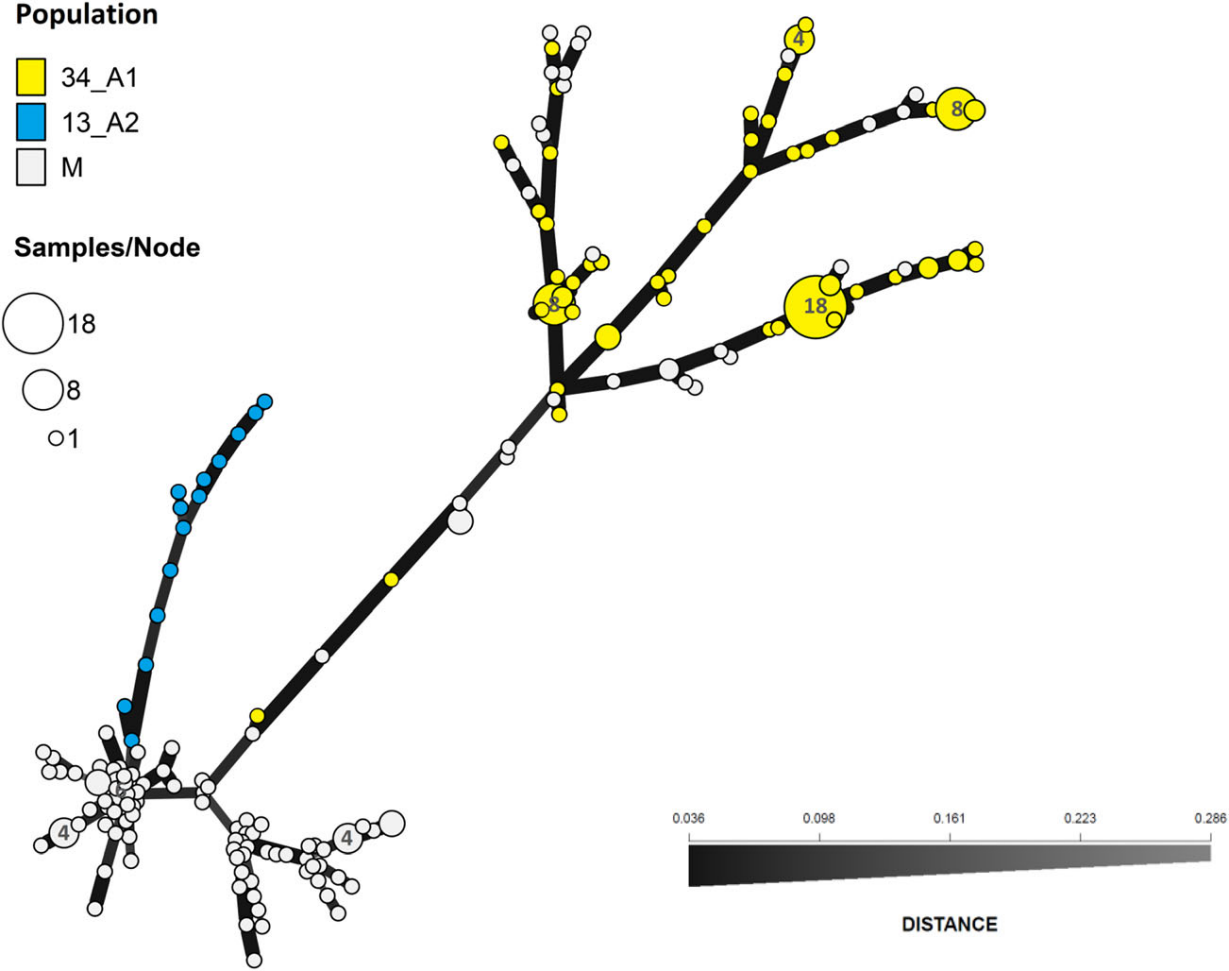
Minimum spanning network of *P. infestans* isolates of 34_A1, 13_A2 and miscellaneous genotypes created from analysis of data from 14 simple sequence repeat markers. Colors indicate genotype membership. Node size is proportional to the number of isolates within the genotype

We distinguished three groups in our *P. infestans* sample. The first group consisted of the isolates of genotype 34_A1 that were all of the A1 mating type and Ia mitochondrial haplotype. They showed diverse reactions to metalaxyl. Out of the 82 isolates tested of genotype 34_A1, 68 were sensitive (83%), 10 were intermediately resistant (12%), and 4 were resistant (5%). Isolates that were intermediately resistant to metalaxyl and those that were resistant did not differ in SSR allele profiles from the isolates that were sensitive to metalaxyl. The second distinct group of isolates contained 14 isolates of genotype 13_A2 that were all of the A2 mating type and Ia mitochondrial haplotype but varied in reaction to metalaxyl: 11 isolates were resistant, and 3 were intermediately resistant. An MSN based on Bruvo’s distance was used to visualize the relationships among isolates of 34_A1, 13_A2, and miscellaneous genotypes (Fig. 4). Large subclonal variation within the 34_A1 genotype was observed in comparison to genotype 13_A2 (Fig. 4).

**Fig. 5:**
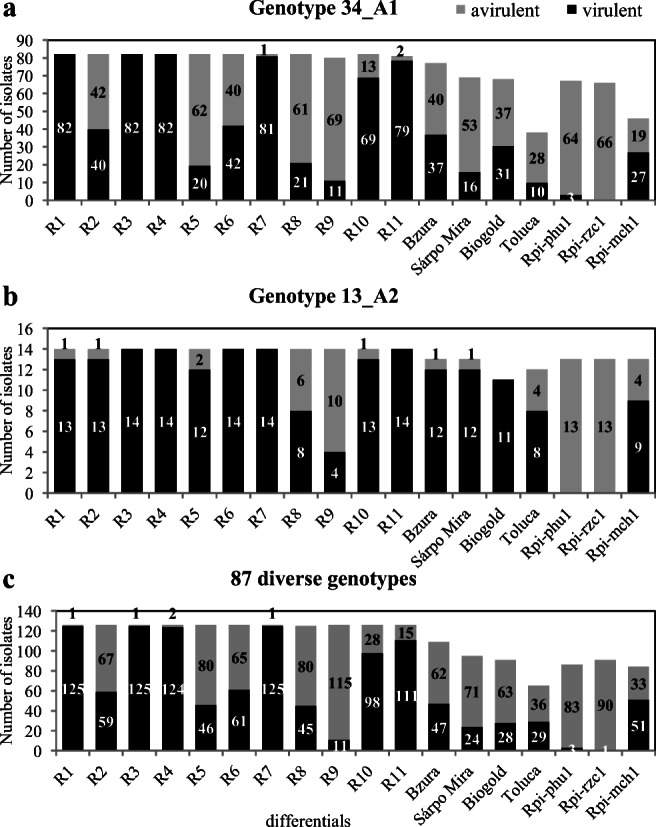
Virulence of *P. infestans* isolates of genotypes 34_A1 (*N* = 82) (**a**), 13_A2 (*N* = 14) (**b**) and 87 diverse genotypes (*N* = 126) (**c**) based on the detached leaflet assay performed with Black’s differential set (R1–R11) and seven other differentials: cvs. Bzura, Sárpo Mira, Biogold, and Toluca and potato clones with the *Rpi-phu1*, *Rpi-rzc1*, and *Rpi-mch1* genes

According to the Mann-Whitney *U* test, differences in the frequency of virulence factors against the set of *R-gene* differentials, including chosen cultivars and new resistance sources, were observed between the 82 isolates of genotype 34_A1 and 14 isolates of genotype 13_A2. Isolates of genotype 13_A2 were more often virulent against the differentials with the *R2*, *R5*, and *R6* genes and the potato cultivars Bzura, Sárpo Mira, Biogold, and Toluca than isolates of 34_A1 (Fig. 5a). Three isolates of *P. infestans* of genotype 34_A1 obtained from plants with *Rpi-phu1* gene were able to overcome the resistance conferred by the gene *Rpi-phu1*, while none of the isolates of genotype 13_A2 (Fig. 5b) were able to do so. None of the isolates of the genotypes 34_A1 and 13_A2 were virulent against the plants with the gene *Rpi-rzc1*. Nearly all isolates of genotypes 34_A1 and 13_A2 were virulent against plants with the *R1*, *R3*, *R4*, *R7*, *R10*, and *R11* genes. Within the isolates of genotype 34_A1, virulence factors against 11 Black’s differentials and new resistance sources showed higher variability than those in the much smaller 13_A2 group. Half of the isolates were virulent toward plants with the *R2* and *R6* genes (40 and 42 isolates, respectively, out of the 82 tested) and toward the cultivars Bzura (37 isolates from 77) and Biogold (31 isolates from 37). In the third group, which consisted of the remaining 87 *P. infestans* genotypes represented by 131 isolates, the isolates of the A1 mating type (69%) and Ia mitochondrial haplotype (68%) and those sensitive to metalaxyl (80%) dominated. The virulence of the 126 isolates was as follows: 88 to 99% of the isolates were able to overcome the resistance conferred by the genes *R1*, *R3*, *R4*, *R7*, and *R11*; 61 to 78% of the isolates were virulent toward plants with the *R10* and *Rpi-mch1* genes; between 25 and 48% of the isolates were virulent toward plants with the *R2*, *R5*, *R6*, and *R8* genes and toward the cultivars Bzura, Sárpo Mira, Biogold, and Toluca; and minimal numbers of isolates were virulent against plants with the *R9* (9%), *Rpi-phu1* (3%), and *Rpi-rzc1* (1%) genes (Fig. 5c).

### Effects of Weather on *P. infestans* Population Structure

Meteorological data were analyzed in two periods each year: the winter season (December, January, February), affecting the overwintering of the inoculum, and summer (June, July, August), shaping the actual disease development. Our hypothesis that mild winters support the spread of *P. infestans* clonal lineages could not be confirmed on the basis of the gathered data. Out of the ten studied winter periods, the lowest average temperature (− 3.7 °C), lowest minimum temperature (− 24.6 °C), and the largest number of days below 0 °C (67 days) were recorded in 2006/2007 (Table 2). After the winter of 2007, only seven isolates of *P. infestans* were collected, and all of these isolates belonged to genotype 34_A1. The second winter period with a low average temperature (− 3.0 °C), low minimum temperature (− 19.2 °C), and a high number of days below 0 °C (63 days) was noted in 2010/2011. Among the 22 *P. infestans* isolates collected in 2011, 17 belonged to genotype 34_A1. After the moderately cold winter seasons of 2009/2010, 2011/2012, and 2013/2014 (Table 2), *P. infestans* isolates of genotype 34_A1 were not observed in large numbers (3, 0, and 1 isolates in 2010, 2012, and 2014, respectively). The winter period of 2007/2008 was the mildest, with the maximum temperature (10.5 °C), a positive average temperature (2.1 °C), and only 20 days with temperatures below 0 °C. In 2008, the highest number of *P. infestans* isolates of genotype 34_A1 was observed (26 isolates). The next mild winter with a positive average temperature (0.6 °C) and a low number of days below 0 °C (35 days) was in 2008/2009. In 2009, a majority of the isolates were of genotype 34_A1 (13 isolates out of 18). In the summer, the highest maximum temperature (28.0 °C), average temperature (19.3 °C), and minimum temperature (13.4 °C) were recorded in 2007 (Table 1), when the number of collected *P. infestans* isolates was the lowest. As shown in Table 1, the highest amount of precipitation during a growing season was noted in 2010 (699.4 mm). The driest year, with 285.5 mm of precipitation, was 2012, when the isolates of genotype 34_A1 were not present.

## Discussion

In this study, the population structure of *P. infestans* isolates collected from one experimental, unprotected field in Boguchwała was analyzed over a long period to check the impact of weather conditions (cold winters, dry and hot summers) on the genetic diversity of the pathogen in different years. Temperature, humidity, and precipitation are the factors that affect different stages of late blight development and survival from year to year. Our hypothesis that cold winters inhibit the spread of clonal lineages and increase the number of diverse *P. infestans* isolates was not confirmed by the obtained data. The genotyping results of SSR markers showed that *P. infestans* isolates of genotype 34_A1 survived at least eight subsequent seasons (Fig. 2). Even after the coldest winter in 2006/2007 and the second coldest in 2010/2011, isolates of genotype 34_A1 were detected. In 2012, we did not observe isolates of genotype 34_A1, although the 2011/2012 winter was not particularly severe, with a rather average temperature. The amount of precipitation during the growing season was not correlated with the incidence of late blight in the field and severity of the disease. In the driest year, 2012, a moderate number of *P. infestans* [27] isolates were collected. We observed no statistically significant differences between samples of *P. infestans* collected in different years in terms of mating type, mitochondrial haplotype, or resistance to metalaxyl. The increase in the number of *P. infestans* isolates resistant to metalaxyl in 2014 in comparison to previous samples was associated with the frequent occurrence of the 13_A2 genotype (Fig. 1). Data from most of the years showed the absence of genetic differentiation of the pathogen. Nei’s genetic identity, F_ST_ values, and Shannon’s Diversity Index corresponded well with each other and indicated a low diversity of *P. infestans* between years of sampling, despite varying weather.

The impact of changing weather conditions on the survival of *P. infestans* isolates as well as on the start and progression of late blight epidemics has been analyzed by many researchers, but there is little data on the effect of weather on the genetic structure of the pathogen population. Brurberg et al. [50] and Grönberg et al. [51] have shown that weather conditions, mainly cold winters, can significantly reduce the survival rate of *P. infestans* mycelia from season to season in plant debris, volunteer potatoes, and weed hosts. In long-term research, Hannukkala et al. [52] investigated the impact of climate and the presence of soil-borne inocula of *P. infestans* in Finland. They monitored late blight symptoms in comparison to weather changes in experimental trials and revealed that epidemics of late blight started earlier and were more intense in the years 1996–2002 than in the historical period 1933–1962. This could be associated with both the occurrence of oospores as a new primary source of inoculum and the increased frequency of precipitation and higher early-season temperature [52]. *Phytophthora infestans* populations from Nordic countries reproduce sexually and are genetically diverse, but recently, researchers observed the appearance of the new invasive clonal lineage EU_41_A2, which is spreading in these populations (EuroBlight network, www.euroblight.net). Additionally, a long-term analysis of potato late blight epidemics in the Netherlands carried out by Zwankhuizen and Zadoks [53] indicated that one single weather factor cannot be responsible for the variation in late blight epidemics. According to the authors, the weather factors that enhance disease development are the number of days with precipitation, number of hours with temperatures between 10 and 27 °C, and air humidity > 90%. The authors also indicated the effects of host, pathogen, and human factors as being very important, particularly crop management and behavior of growers regarding fungicides.

The set of *P. infestans* isolates analyzed in this study allowed us to assess the intragenotype diversity of the genotypes 34_A1 and 13_A2 to some extent. The genotype 34_A1 was the most frequent, represented by 87 isolates out of 237 tested *P. infestans* isolates. Previous data on the *P. infestans* population structure in Poland have indicated that sexual reproduction is likely occurring in this population [16]. The STRUCTURE analysis of the data from Boguchwała showed that some of the *P. infestans* isolates were grouped simultaneously into three or two clusters (Fig. 3a, b). We presumably observed some isolates that are a consequence of sexual reproduction of isolates of the 34_A1 genotype (partially belonging to the green cluster) rather than a result of mutation. In each year of research, *P. infestans* isolates of both mating types were observed. The genotype 34_A1 was first recorded in 2004 in Slovenia (EuroBlight network, www.euroblight.net). In Poland, the earliest isolates of this genotype were detected in 2002 (two isolates). In the present study, we found 14 isolates of the second well-known genotype of *P. infestans*, 13_A2, which rapidly spread throughout Europe between 2005 and 2008 [8] and was represented previously by only a few isolates in Poland [15]. In spite of seed potato imports from EU to Poland (between 2005 and 2014 the import fluctuated between 11.7 and 33 thousands of tons), the 13_A2 *P. infestans* genotype remains rather rare. These two genotypes differed in virulence factors (Fig. 5a, b) and resistance to metalaxyl.

According to Cooke et al. [8], isolates of the genotype 13_A2 are resistant to metalaxyl. There are no data on the metalaxyl resistance of 34_A1 in the literature. Another known genotype, EU_33_A2 (Green 33), has been described as having reduced sensitivity to a fungicide (fluazinam), and researchers recommend reducing the number of fluazinam sprays in *P. infestans* populations in which isolates of this genotype occur [54].

The experimental field was not sprayed with metalaxyl or any other fungicide in any year of *P. infestans* collection. We observed *P. infestans* isolates that were sensitive, intermediately resistant, and resistant to metalaxyl within the genotype 34_A1. *P. infestans* isolates within the genotype 13_A2 were intermediately resistant or resistant to metalaxyl, but distinguishing between those two categories may be difficult. For this reason, strategies for efficient control of late blight based solely on knowledge of a *P. infestans* genotype could be insufficient and imprecise, although the resistance assessment could also be incorrect, especially between the intermediately resistant and resistant categories. We also observed differences in virulence within isolates of the same genotype (Fig. 5a, b), which illustrates how difficult it is to draw conclusions about the invasive potential of pathogen genotypes and control late blight.

Another explanation for the observed diversity within SSR genotypes could be incorrect genotype calling and assignment. This is especially difficult in populations where sexual recombination may be occurring and where boundaries between recombinants and genotype variants arising through mutation are not clear. We allowed up to two changed alleles within a genotype. As a result, we observed in the MSN isolates classified as miscellaneous genotypes on the same edges as isolates of the 34_A1 genotype (Fig. 4). Sixteen of those miscellaneous isolates would have been included in the 34_A1 genotype, if we assigned the differences in four alleles as variants of one genotype (personal communication David Cooke), but the miscellaneous isolates mixed up on the same edges with the 34_A1 isolates would not be eliminated completely (Supplementary Table S1). It is likely that some isolates of miscellaneous genotypes clustered together with isolates of the 34_A1 genotype are products of the sexual reproduction that takes place in Poland.

All of these results provide important insights into the genetic diversity and structure of local *P. infestans* populations over a long timescale with regard to weather conditions. We reported the occurrence of many isolates of the genotype 34_A1 in one unprotected experimental field in Boguchwała in the southwestern part of Poland. We also observed the survival of *P. infestans* isolates of a single genotype in consecutive years despite harsh conditions, with the lowest average temperature (− 3.7 °C), lowest minimum temperature (− 24.6 °C), and highest number of days with temperatures below 0 °C (67) in the winter season. This observation might be meaningful for the effective control of late blight and management of crop rotation.

## Electronic supplementary material

ESM 1 (XLSX 67 kb)

ESM 2 (PDF 207 kb)

ESM 3 (XLSX 36 kb)

ESM 4 (XLSX 14 kb)

## Data Availability

Selected pure cultures of *P. infestans* are maintained and available from Plant Breeding and Acclimatization Institute—National Research Institute in Poland.
