# Supplementary figures and images for: Population Structure of Phytophthora infestans from a Single Location in Poland Over a Long Period of Time in Context of Weather Conditions
Source: Microb Ecol. 2020 Oct 29;81(3):746–57. doi: 10.1007/s00248-020-01630-6 (PMC7982385; doi:10.1007/s00248-020-01630-6)

## Virulence of 226 Polish *P. infestans* isolates

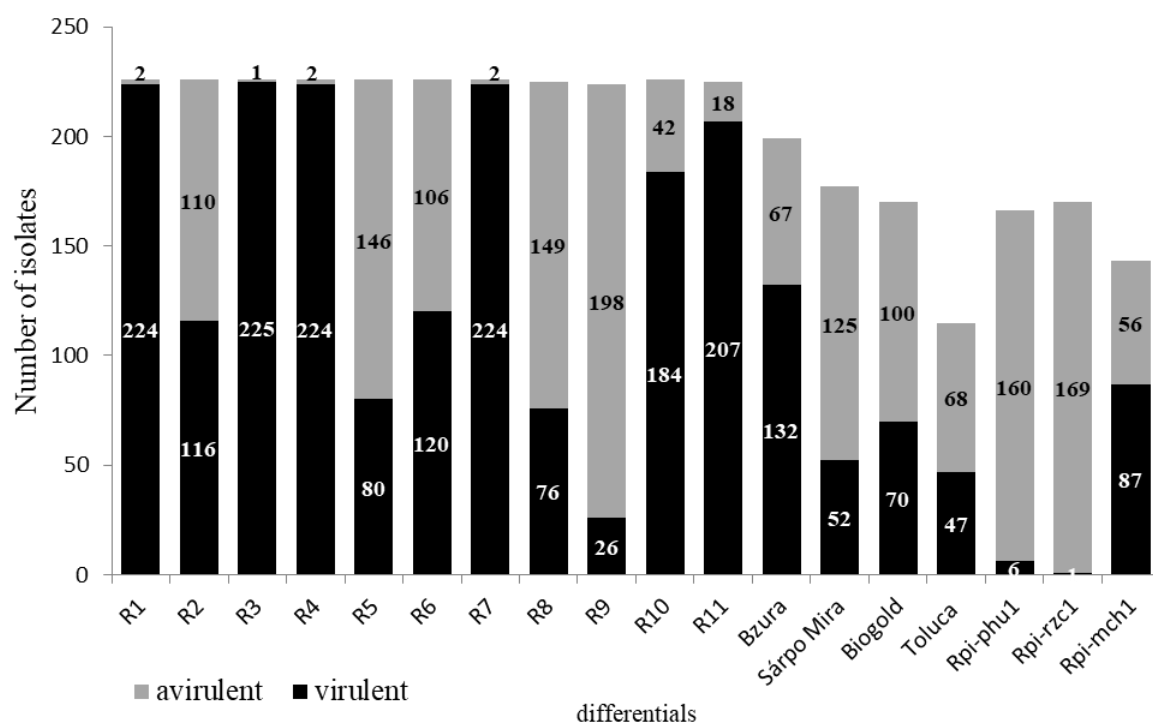

Supplement: Supplementary file 2 — (PDF 207 kb) [file 248_2020_1630_MOESM2_ESM.pdf]
